# Impact of Long-Term HFD Intake on the Peripheral and Central IGF System in Male and Female Mice

**DOI:** 10.3390/metabo10110462

**Published:** 2020-11-13

**Authors:** Santiago Guerra-Cantera, Laura M. Frago, María Jiménez-Hernaiz, Purificación Ros, Alejandra Freire-Regatillo, Vicente Barrios, Jesús Argente, Julie A. Chowen

**Affiliations:** 1 Department of Endocrinology, Hospital Infantil Universitario Niño Jesús, Instituto de Investigación La Princesa, E-28009 Madrid, Spain; santiago.guerra@estudiante.uam.es (S.G.-C.); laura.frago@uam.es (L.M.F.); maria_jhernaiz@hotmail.es (M.J.-H.); alefreirefr@gmail.com (A.F.-R.); vicente.barriossa@salud.madrid.org (V.B.); 2 Department of Pediatrics, Faculty of Medicine, Universidad Autónoma de Madrid, E-28029 Madrid, Spain; prosmon@hotmail.com; 3 Centro de Investigación Biomédica en Red de Fisiopatología de la Obesidad y Nutrición (CIBEROBN), Instituto de Salud Carlos III, E-28029 Madrid, Spain; 4 Department of Pediatrics, Hospital Universitario Puerta de Hierro-Majadahonda, E-28222 Madrid, Spain; 5 IMDEA Food Institute, CEI UAM + CSIC, Carretera de Cantoblanco 8, E-28049 Madrid, Spain

**Keywords:** IGF1, IGF2, IGFBP2, high-fat diet, obesity, sex differences, neuropeptides

## Abstract

The insulin-like growth factor (IGF) system is responsible for growth, but also affects metabolism and brain function throughout life. New IGF family members (i.e., pappalysins and stanniocalcins) control the availability/activity of IGFs and are implicated in growth. However, how diet and obesity modify this system has been poorly studied. We explored how intake of a high-fat diet (HFD) or commercial control diet (CCD) affects the IGF system in the circulation, visceral adipose tissue (VAT) and hypothalamus. Male and female C57/BL6J mice received HFD (60% fat, 5.1 kcal/g), CCD (10% fat, 3.7 kcal/g) or chow (3.1 % fat, 3.4 kcal/g) for 8 weeks. After 7 weeks of HFD intake, males had decreased glucose tolerance (*p <* 0.01) and at sacrifice increased plasma insulin (*p <* 0.05) and leptin (*p <* 0.01). Circulating free IGF1 (*p <* 0.001), total IGF1 (*p <* 0.001), IGF2 (*p <* 0.05) and IGFBP3 (*p <* 0.01) were higher after HFD in both sexes, with CCD increasing IGFBP2 in males (*p <* 0.001). In VAT, HFD reduced mRNA levels of IGF2 (*p <* 0.05), PAPP-A (*p <* 0.001) and stanniocalcin (STC)-1 (*p <* 0.001) in males. HFD increased hypothalamic IGF1 (*p <* 0.01), IGF2 (*p <* 0.05) and IGFBP5 (*p <* 0.01) mRNA levels, with these changes more apparent in females. Our results show that diet-induced changes in the IGF system are tissue-, sex- and diet-dependent.

## 1. Introduction

The insulin-like growth factor (IGF) system has been widely studied in both pre- and postnatal growth and development, but this system is involved in a myriad of functions throughout life with much less known regarding many of these diverse roles. Members of this family include the ligands IGF1 and IGF2, which are crucial for longitudinal bone growth [1] and both also have additional important functions throughout life. Studies of IGF2 have focused mainly on its fundamental role during fetal life, as its expression decreases in multiple organs during aging in mice [2] as well as in the circulation in humans [3], but less is known about its postnatal actions. IGF1 and IGF2 bind to six different IGF binding proteins (IGFBPs) to increase their half-life and for transport to target tissues and importantly, to reduce their potential hypoglycemic effect. IGFBP3 is the most abundant binding protein in circulation, carrying from 70% to 90% of circulating IGF1 and 2 [4]. When IGF1 or IGF2 are bound to IGFBP3 or IGFBP5, it also binds to an acid-labile subunit (ALS) forming a trimolecular 150 KDa complex [5]. It is estimated that around 80% of the IGFs are bound in these trimolecular complexes, approximately 20% are bound to other IGFBPs and less than 1% are in the free form [6]. IGFBP2 is the second most abundant binding protein in serum, at least in some conditions [7,8], with a slight preference for IGF2 over IGF1 [9]. However, expression levels of the IGFBPs are tissue-specific, with, for example, IGFBP2 being the most abundant IGFBP in the postnatal central nervous system (CNS) [10], as well as in white preadipocytes during adipogenesis [11]; while IGFBP4 is the most abundant in cultured adult human adipose tissue [12].

When released from their binding proteins, the IGFs can activate the IGF1 receptor (IGF1R), which is structurally similar to the insulin receptor and when phosphorylated activates the PI3K/AKT pathway. Activation of this pathway underlies the anabolic effects of the IGFs [13]. In contrast, IGF2R is classified as a scavenger receptor for removing excess IGF2 from the circulation linked to lysosomal enzymes [14], with IGF2 having a 100-fold higher binding affinity for the IGF2R than IGF1 does [14].

New members of this system identified in recent years include pregnancy-associated plasma protein-A (PAPP-A) and PAPP-A2. These pappalysins modulate the biological activity of IGFs by cleaving the ligand from the binding protein and thus allowing them to bind to their receptors [15]. In addition, stanniocalcin (STC)-1 and 2 (STC-2) inhibit the proteolytic activity of pappalysins, reducing the release of IGF1 and 2 from the IGFBPs, hence their binding receptors [16,17].

The IGF system is important in anabolism, causing muscle hypertrophy [18], and is crucial in adipocyte maturation and differentiation [19]. Moreover, it can be altered by nutritional status [20]. In the brain it is important during development, but also throughout life. Indeed, in addition to peripheral IGF1 and IGF2 crossing the blood–brain barrier to reach the brain [21,22], IGFs are locally produced by brain cells, especially microglia and astrocytes [23,24]. These centrally produced IGFs can act as neuroprotective factors during events that result in gliosis and neuroinflammation. In the CNS, IGF1 is involved in postnatal synaptogenesis and neurogenesis [25], amyloid clearance [26], protection against neuroinflammation [24] and in neuroprotection [27], as well as with regulation of brain glucose metabolism [28]. The functions of IGF2 in the postnatal brain have been studied less, despite the fact that, for example, it is highly expressed in the hippocampus and has been associated with memory consolidation [29].

Gliosis, involving both astrocytes and microglia, is initially a protective response [30,31] but when prolonged can become damaging [32]. In response to high-fat diet (HFD) consumption, astrocytes are thought to participate in both the early protective response [33] and the detrimental effects of prolonged HFD intake and obesity [34]. The neuroprotective actions of astrocytes against oxidative stress [27], apoptosis and inflammation [35] are executed at least partially through the release of IGF1 [36,37] and consequently the downstream activation of the pro-survival pathway PI3K [38]. Thus, it is possible that the IGF system is involved in neuroprotection against the noxious effects of poor nutrition.

We have previously reported that short-term dietary changes modify the IGF system, with both a HFD and commercial control diet (CCD) inducing sex-specific changes peripherally and centrally compared to a normal rodent chow diet [39]. Here, our objective was to evaluate the long-term effects of HFD and CCD consumption on the central and peripheral IGF systems in male and female mice.

## 2. Results

### 2.1. Body Composition

**Figure 1:**
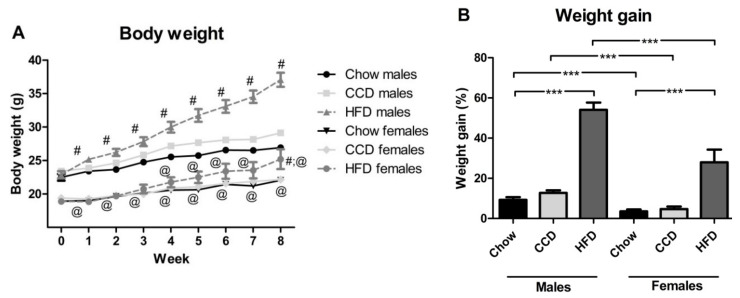
Body weight progression (**A**) and accumulated weight gain in percentage (**B**) in male and female mice exposed to a high-fat diet (HFD), commercial control diet (CCD) or a chow diet for 8 weeks. *** *p <* 0.001; #: different from chow in the same sex, @: different between sexes on the same diet. *n* = 6.

Body weight changed with time throughout the study (F_(8,53)_ = 325.6, *p <* 0.001; Figure 1A),and was also influenced by sex (F_(1,53)_ = 235.6, *p <* 0.001) and diet (F_(2,53)_ = 19.4, *p <* 0.001). At study onset, there were differences between the sexes (F_(1,53)_ = 27.4, *p <* 0.001) in body weight (Figure 1A), with males weighing more than females and these sex differences were maintained throughout the study. HFD increased body weight in males from the first week (F_(2,26)_ = 5.2, *p <* 0.05) until the last week of the study (F_(2,26)_ = 62.2, *p <* 0.001). However, in females HFD intake induced a significant increase in weight only at the last week of the study (F_(2,26)_ = 4.0, *p <* 0.05).

Weight gain (Figure 1B) was influenced by both sex (F_(1,53)_ = 27.4, *p <* 0.001), with males gaining more weight than females, and diet (F_(2,53)_ = 77.2, *p <* 0.001), with an interaction between these factors (F_(2,53)_ = 6.4, *p <* 0.01). HFD induced weight gain in both males (F_(2,26)_ = 116.2, *p <* 0.001) and females (F_(2,26)_ = 13.5, *p <* 0.001).

Body weight at sacrifice (Table 1) was determined by sex (F_(1,53)_ = 134.5, *p <* 0.001), with males weighing more than females, and by diet (F_(2,53)_ = 55.4, *p <* 0.001), with an interaction between these two factors (F_(2,53)_ = 8.5, *p <* 0.01). Body weight was higher after HFD consumption in both males (F_(2,26)_ = 72.2, *p <* 0.001) and females (F_(2,26)_ = 8.2, *p <* 0.01), with no effect of CCD intake on final body weight.

The amount of visceral adipose tissue (VAT; Table 1) was affected by both sex (F_(1,53)_ = 24.8, *p <* 0.001), with males having higher levels than females, and diet (F_(2,53)_ = 60.7, *p <* 0.001), with an interaction between these factors (F_(2,53)_ = 9.1, *p <* 0.001). HFD intake increased the percentage of VAT in both males (F_(2,26)_ = 94.9, *p <* 0.001) and females (F_(2,26)_ = 8.6, *p <* 0.01). Subcutaneous adipose tissue content (Table 1) was influenced by diet (F_(2,53)_ = 6.3, *p <* 0.001), with an interaction between sex and diet (F_(2,53)_ = 4.9, *p <* 0.05). The percentage of subcutaneous adipose tissue was increased by HFD intake in both males (F_(2,26)_ = 58.4, *p <* 0.001) and females (F_(2,26)_ = 10.5, *p <* 0.01).

The number of kcal ingested per day was influenced by sex (F_(1,17)_ = 62.5, *p <* 0.001; Table 1) and diet (F_(2,17)_ = 267.9, *p <* 0.001). There was also an interaction between sex and diet (F_(2,17)_ = 135.9, *p <* 0.001), with HFD only increasing energy intake per mouse in females (F_(2,8)_ = 331.0, *p <* 0.001). When adjusted for body weight, females consumed more energy than males (F_(1,17)_ = 232.4, *p <* 0.001), with this also being affected by diet (F_(2,17)_ = 255.5, *p <* 0.001). There was an interaction between sex and diet (F_(2,17)_ = 232.4, *p <* 0.001) as energy intake/g body weight was only increased by HFD in females (F_(2,8)_ = 331.0, *p <* 0.001).

Energy efficiency, determined as the amount of weight gained per kilocalories consumed, was higher in males compared to females (F_(1,17)_ = 156.4, *p <* 0.001; Table 1). Energy efficiency was also influenced by diet (F_(2,17)_ = 37.3, *p <* 0.001), with an interaction between sex and diet (F_(2,17)_ = 51.9, *p <* 0.001). This parameter was higher in males fed a HFD (F_(2,8)_ = 63.2, *p <* 0.001) compared to those on chow.

Circulating leptin levels were affected by diet (F_(2,35)_ = 19.8, *p <* 0.01; Table 1) with an interaction between sex and diet (F_(2,35)_ = 4.3, *p <* 0.05). HFD increased plasma leptin levels in both males (F_(2,18)_ = 16.3, *p <* 0.001) and females (F_(2,16)_ = 5.5, *p <* 0.05).

### 2.2. Glucose Tolerance Test and Insulin Levels

Glycemia at sacrifice was affected by diet (F_(2,53)_ = 7.6, *p <* 0.01), with an increase after HFD in both sexes (Table 1).

**Figure 2:**
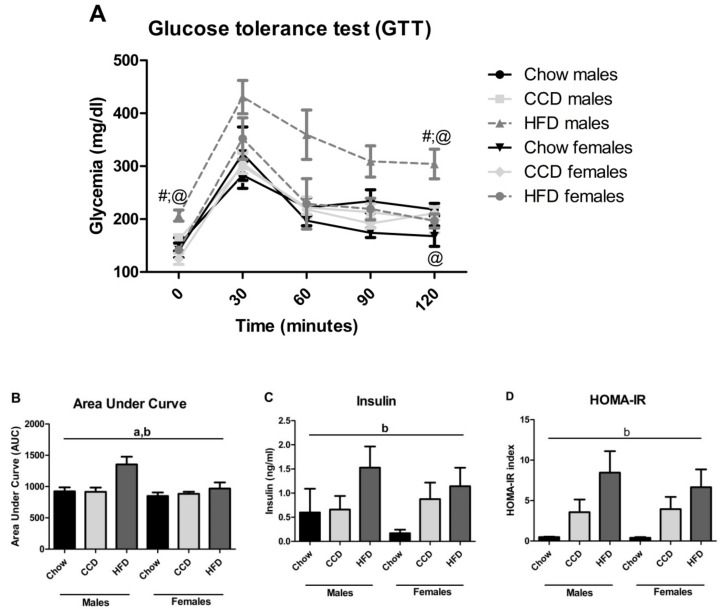
Glucose tolerance test and insulin levels. Glucose levels over time (**A**), area under curve (**B**), plasma insulin levels (**C**) and Homeostatic Model Assessment for Insulin Resistance (HOMA-IR; **D**) in male and female mice on a high-fat diet (HFD), commercial control diet (CCD) or a chow diet for 8 weeks. a: overall effect of sex, b: overall effect of diet, #: different from chow on the same sex, @: differences between sexes on the same diet, *n* = 6.

In the glucose tolerance test (Figure 2A), diet had an overall effect (F_(2,34)_ = 7.9, *p <* 0.01) as did sex (F_(1, 34)_ = 7.8, *p <* 0.01), with an interaction between these factors (F_(2,34)_ = 3.3, *p <* 0.05) and a change over time (F_(4,34)_ = 79.3, *p <* 0.001). Basal glycemia levels showed differences according to sex (F_(1,34)_ = 18.9, *p <* 0.001) and diet (F_(2,34)_ = 5.3, *p <* 0.05), with an interaction between these factors (F_(2,34)_ = 3.2, *p =* 0.05). At baseline, there were sex differences when CCD was consumed (F_(1,11)_ = 6.6, *p <* 0.05) as well as with HFD (F_(1,11)_ = 30.1, *p <* 0.001), with males presenting a higher glycemia than females in both cases.

In males, HFD increased glycemia (F_(2,17)_ = 7.0, *p <* 0.01). At 30 min (F_(2,34)_ = 4.9, *p <* 0.05), 60 min (F_(2,34)_ = 4.5, *p <* 0.05) and 90 min (F_(2,34)_ = 6.6, *p <* 0.01) there was an effect of diet on the response to a glucose bolus with an increase in glycemia after HFD consumption. There was an effect of sex at 60 (F_(1,34)_ = 4.5, *p <* 0.05) and 90 min (F_(1,34)_ = 12.8, *p <* 0.01), with overall higher levels in males than females.

At 120 min, there was an effect of sex (F_(1,34)_ = 9.5, *p <* 0.01) and diet (F_(2,34)_ = 5.0, *p <* 0.05), and an interaction between these two factors (F_(2,34)_ = 5.0, *p <* 0.05). There were differences between males and females on chow (F_(1,10)_ = 5.3, *p <* 0.05) and HFD (F_(1,11)_ = 12.1, *p <* 0.01), with males having increased glycemia in both cases. Males on a HFD had a higher glycemia compared to when consuming chow (F_(2,17)_ = 6.3, *p <* 0.05).

The area under the curve (AUC; Figure 2B) was affected by sex (F_(1,34)_ = 6.2, *p <* 0.05) and diet (F_(2,34)_ = 7.6, *p <* 0.01), with the increase after HFD consumption being more apparent in males.

Plasma insulin levels (F_(2,33)_ = 3.7, *p <* 0.05: Figure 2C) and the Homeostatic Model Assessment for Insulin Resistance (HOMA-IR) (F_(2,31)_ = 7.6, *p <* 0.01; Figure 2D) were affected by diet, with both HFD and CCD inducing an overall increase in these parameters, with the CCD effect on insulin being more apparent in females.

### 2.3. Circulating Levels of the IGF System

**Figure 3:**
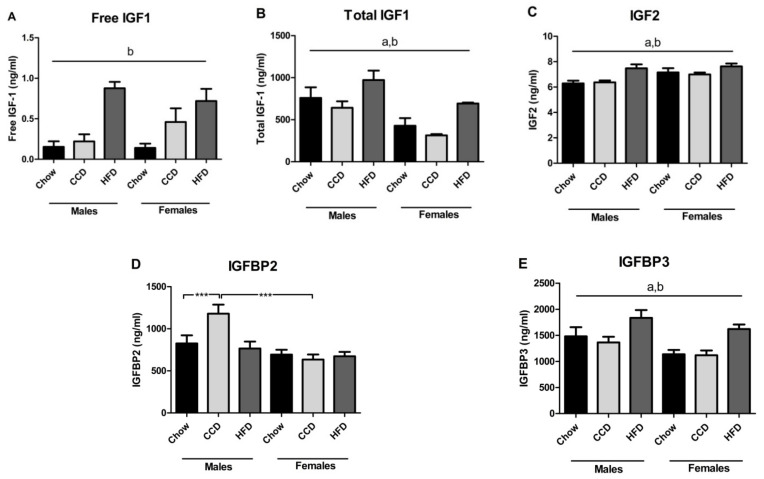
Circulating levels of free IGF-1 (**A**), total IGF-1 (**B**), IGF2 (**C**), IGFBP2 (**D**) and IGFBP3 (**E**) in mice on a high-fat diet (HFD), commercial control diet (CCD) or chow diet for 8 weeks. *** *p <* 0.001, a: effect of sex, b: effect of diet, *n* = 6.

Free IGF1 levels were modified by diet (F_(2,29)_ = 18.6, *p <* 0.001; Figure 3A), with an increase after HFD consumption in both sexes. Total IGF1 was affected by sex (F_(1,34)_ = 19.0, *p <* 0.001; Figure 3B), with males having higher levels than females, and by diet (F_(2,34)_ = 8.2, *p <* 0.01) with an increase after HFD intake in both sexes.

Circulating IGF2 levels were affected by sex (F_(1,35)_ = 7.6, *p <* 0.05; Figure 3C), with females having overall higher levels than males, and diet (F_(2,35)_ = 8.2, *p <* 0.01), with HFD inducing an overall increase in IGF2 levels. There was an effect of sex on plasma IGFBP2 levels (F_(1,35)_ = 15.9, *p <* 0.001; Figure 3D), with males having higher levels of IGFBP2 than females. There was an interaction between sex and diet (F_(2,35)_ = 5.1, *p <* 0.05) with sex differences in IGFBP2 levels after CCD consumption (F_(1,11)_ = 19.6, *p <* 0.01). In males, there was an increase in IGFBP2 after CCD consumption (F_(2,17)_ = 5.5, *p* = 0.05).

There was also an effect of sex (F_(1,35)_ = 7.3, *p <* 0.05), with males having higher levels than females, and diet (F_(2,35)_ = 9.5, *p <* 0.01) on plasma IGFBP3 levels (Figure 3E), with HFD increasing the levels of this binding protein in both sexes.

### 2.4. The IGF System in Visceral Adipose Tissue (VAT)

**Figure 4:**
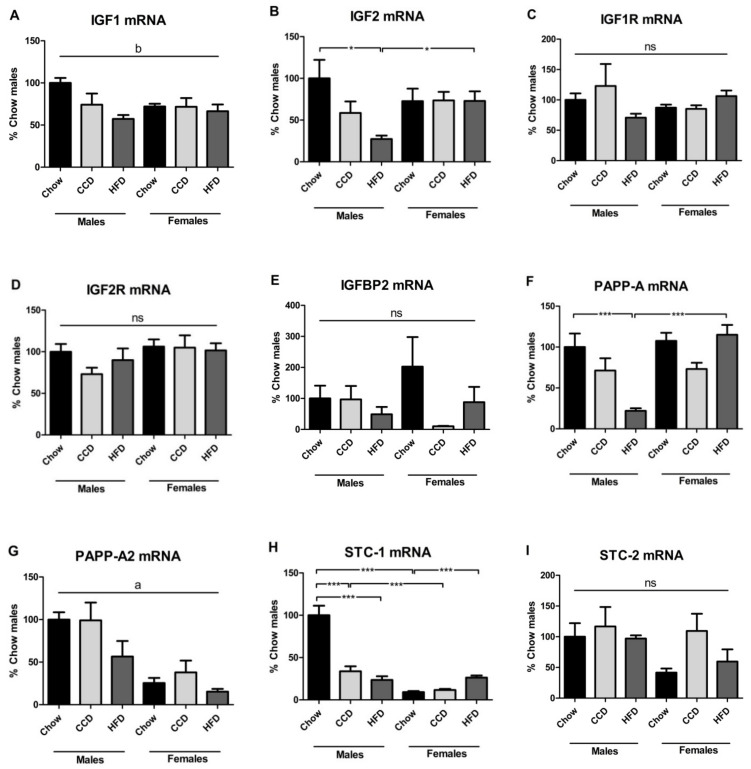
Relative gene expression of the insulin-like growth factor (IGF) system in visceral adipose tissue: IGF1 (**A**), IGF2 (**B**), IGF1R (**C**), IGF2R (**D**), IGFBP2 (**E**), PAPP-A (**F**), PAPP-A2 (**G**), stanniocalcin (STC)-1 (**H**) and STC-2 (**I**) in mice on a high-fat diet (HFD), commercial control diet (CCD) or a chow diet for 8 weeks. * *p <* 0.05, *** *p <* 0.001, a: effect of sex, b: effect of diet, ns = non-significant, *n* = 6.

Relative IGF1 mRNA levels were modified by diet (F_(2,33)_ = 4.5, *p <* 0.05; Figure 4A), with a decrease after HFD in males. There was an interaction between sex and diet regarding IGF2 mRNA levels (F_(2,34)_ = 3.2, *p* = 0.05; Figure 4B). In males, relative IGF2 mRNA levels were decreased after HFD intake (F_(2,16)_ = 4.9, *p <* 0.05), resulting in males having lower levels than females when on a HFD (F_(1,10)_ = 11.9, *p <* 0.01). There was no effect of sex or diet on relative mRNA levels of IGF1R (Figure 4C), IGF2R (Figure 4D) or IGFBP2 (Figure 4E).

Relative PAPP-A mRNA levels were affected by both diet (F_(2,34)_ = 5.3, *p <* 0.05; Figure 4F) and sex (F_(1,34)_ = 12.1, *p <* 0.01), with an interaction between these factors (F_(2,34)_ = 8.8, *p <* 0.01). In males, PAPP-A mRNA levels were reduced after HFD intake (F_(2,16)_ = 7.7, *p <* 0.01), with no HFD effect in females resulting in males having lower levels when on a HFD (F_(1,10)_ = 47.3, *p <* 0.001). Relative PAPP-A2 mRNA levels were only affected by sex (F_(1,33)_ = 28.2, *p <* 0.001; Figure 4G), being higher in males than females.

Relative STC-1 mRNA levels were modified by diet (F_(2,32)_ = 18.2, *p <* 0.001; Figure 4H) and sex (F_(1,32)_ = 58.3, *p <* 0.001), with an interaction between these factors (F_(2,32)_ = 33.8, *p <* 0.001). STC-1 mRNA levels were higher in males than females when on chow (F_(1,10)_ = 52.8, *p <* 0.001) or a CCD (F_(1,10)_ = 16.8, *p <* 0.01). HFD consumption led to decreased levels in males (F_(2,15)_ = 25.8, *p <* 0.001) but increased in females (F_(2,16)_ = 26.2, *p <* 0.001). In males, STC-1 mRNA levels were also decreased after CCD intake (F_(2,15)_ = 25.8, *p <* 0.001). No significant differences in STC-2 mRNA levels were found (Figure 4I).

### 2.5. Hypothalamic Response to Dietary Change

#### 2.5.1. The IGF System in the Hypothalamus

**Figure 5:**
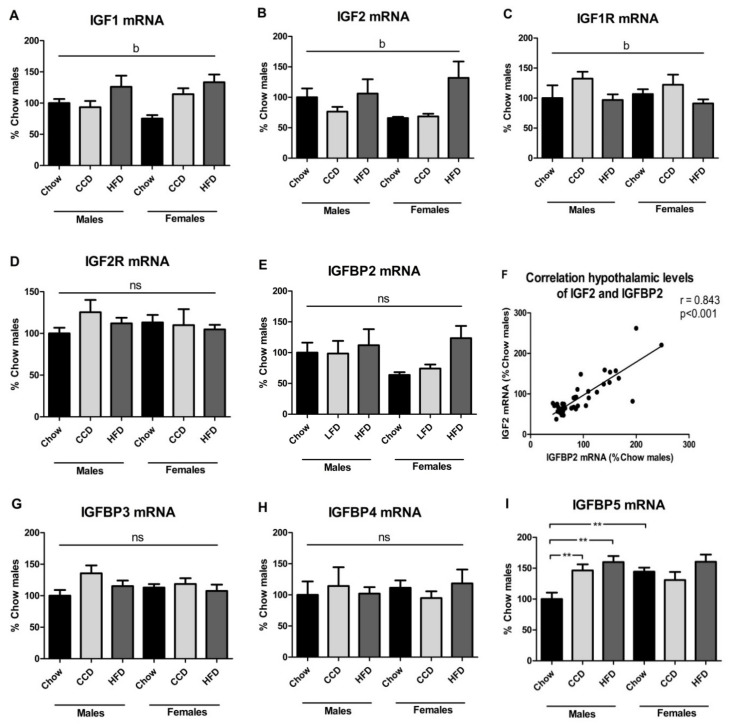

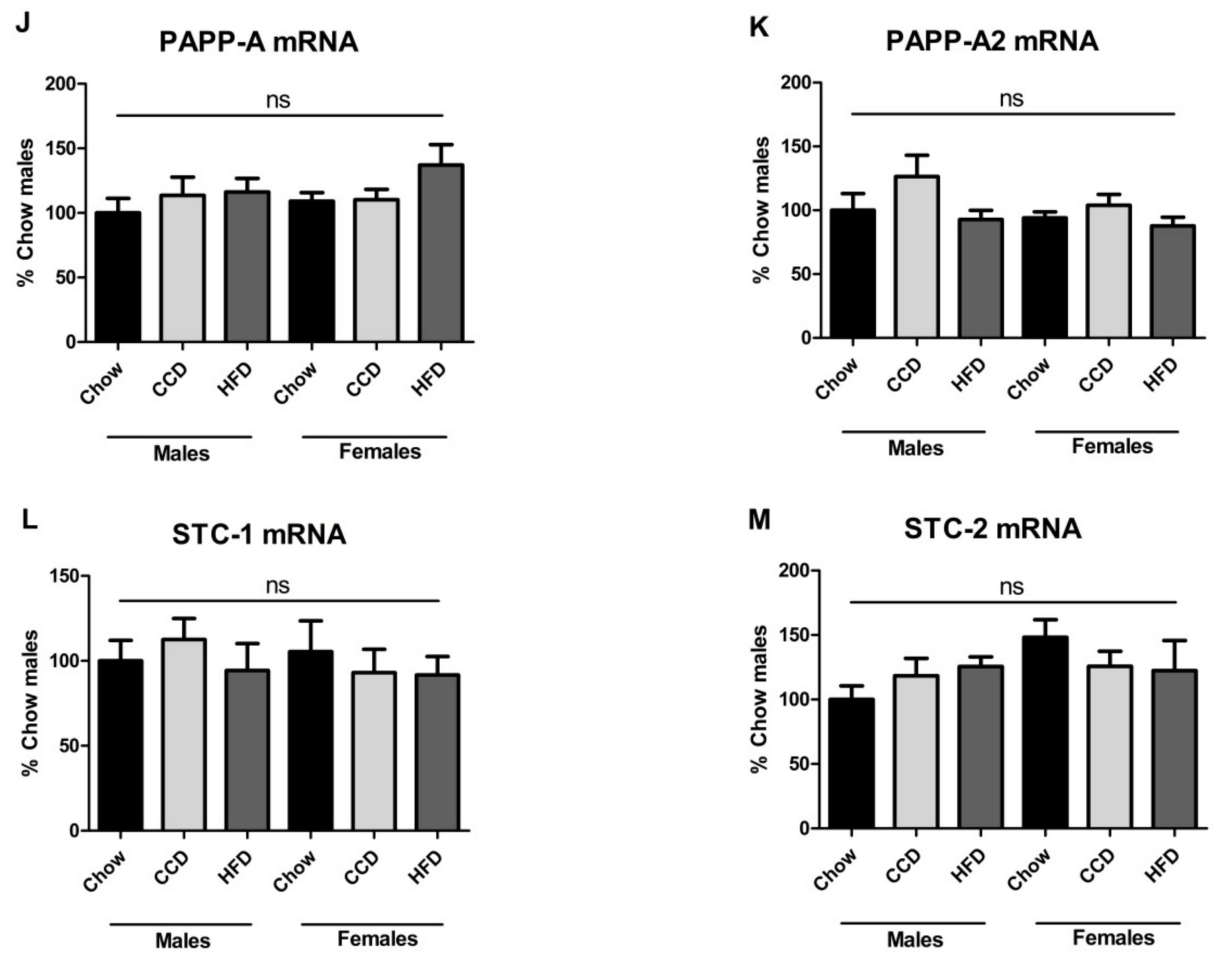
Relative hypothalamic mRNA levels of the IGF system: IGF1 (**A**), IGF2 (**B**), IGF1R (**C**), IGF2R (**D**), IGFBP2 (**E**), IGFBP3 (**G**), IGFBP4 (**H**), IGFBP5 (**I**), PAPP-A (**J**), PAPP-A2 (**K**), STC-1 (**L**) and STC-2 (**M**). Correlation of relative hypothalamic mRNA levels of IGF2 and IGFBP2 (**F**). ** *p <* 0.01, b: effect of diet, ns = non-significant, HFD = high-fat diet, CCD = commercial control diet, *n* = 6.

The hypothalamic levels of IGF1 mRNA were affected by diet (F_(2,43)_ = 6.8, *p <* 0.01; Figure 5A), being increased after HFD consumption in both sexes. There was also an effect of diet on the hypothalamic mRNA levels of IGF2 (F_(2,42)_ = 4.7, *p <* 0.05; Figure 5B), with an overall increase in response to HFD, with this being more apparent in females. Relative IGF1R mRNA levels were altered by diet (F_(2,35)_ = 3.3, *p* = 0.05; Figure 5C) with an overall increase in response to the CCD. However, there were no effects of either sex or diet on relative IGF2R mRNA levels (Figure 5D).

Regarding IGFBP2 mRNA expression, despite the apparent increase after HFD consumption in females, there was no overall effect of sex or diet (Figure 5E). As previously reported [39], the relative mRNA levels of IGF2 and IGFBP2 showed a similar profile, with a significant positive correlation between these two factors (*r* = 0.843, *p <* 0.001; Figure 5F).

We observed no differences in the relative mRNA levels of IGFBP3 (Figure 5G) or IGFBP4 (Figure 5H). However, IGFBP5 mRNA levels were affected by diet (F_(2,35)_ = 6.8, *p <* 0.01; Figure 5I) with an interaction between sex and diet (F_(2,35)_ = 4.5, *p <* 0.05). When on a chow diet, females were found to have higher levels than males (F_(1,11)_ = 13.1, *p <* 0.01). When separated by sex, CCD and HFD consumption were found to increase the mRNA levels of IGFBP5 in the hypothalamus in males (F_(2,17)_ = 10.0, *p <* 0.01), with no significant effect of diet in females.

Relative hypothalamic mRNA levels of PAPP-A (Figure 5J), PAPP-A2 (Figure 5K), STC-1 (Figure 5L) and STC-2 (Figure 5M) were not affected by sex or diet.

IGFBP2 is proposed to be an antidiabetic factor, protecting against obesity onset [40,41]. In addition, taking into consideration that IGFBP2 has preference for IGF2 over IGF1 [9], we determined the statistical correlations between these factors in the circulation, VAT, and the hypothalamus with body weight and glycemia (Table 2). Plasma IGF2 was positively and IGFBP2 negatively correlated with body weight, but exclusively in males. No significant correlations were found with glycemia in either sex. In VAT, the only correlation observed was that relative IGF2 mRNA levels were negatively correlated with body weight in males. In the hypothalamus there was a positive correlation between IGF2 mRNA levels and both body weight and glycemia, but only in females. Hypothalamic IGFBP2 mRNA relative levels also correlated positively with both body weight and glycemia and again only in females.

#### 2.5.2. Hypothalamic Neuropeptides

**Figure 6:**
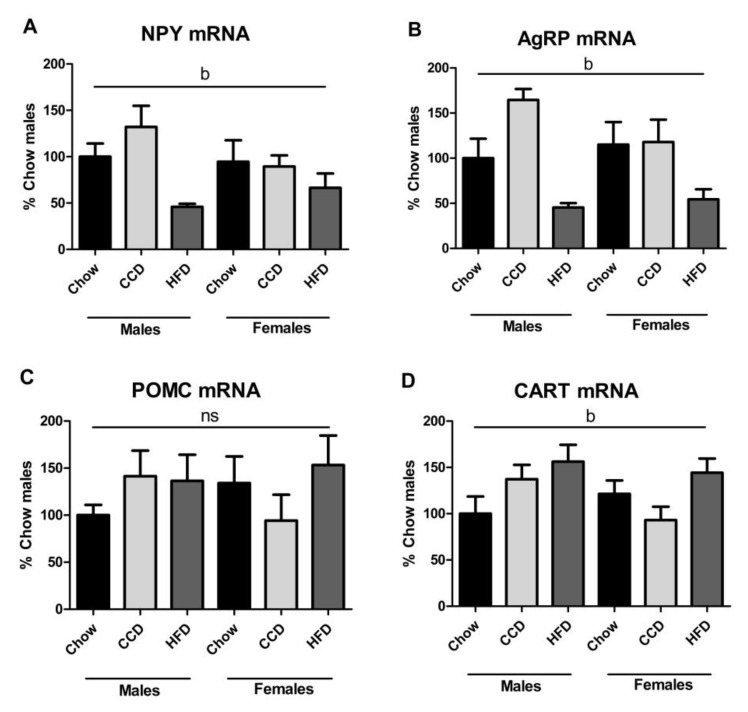
Relative mRNA levels of orexigenic and anorexigenic neuropeptides in the hypothalamus: Neuropeptide Y (NPY; **A**), Agouti-related protein (AgRP; **B**), proopiomelanocortin (POMC; **C**) and cocaine and amphetamine regulated transcript (CART; **D**). b: effect of diet, ns = non-significant. HFD = high-fat diet; CCD = commercial control diet, *n* = 6.

The relative mRNA levels of neuropeptide Y (NPY) (F_(2,35)_ = 5.8, *p <* 0.01; Figure 6A) and Agouti-related protein (AgRP) (F_(2,34)_ = 11.9, *p <* 0.001; Figure 6B) in the hypothalamus were affected by diet, with the mRNA levels of both orexigenic neuropeptides decreasing after HFD intake.

Proopiomelanocortin (POMC) mRNA levels (Figure 6C) were not altered by sex or diet, whereas relative cocaine and amphetamine regulated transcript (CART) expression was affected by diet (F_(2,35)_ = 3.6, *p <* 0.05; Figure 6D), with HFD inducing an overall increase.

#### 2.5.3. Gliosis and Hypothalamic Stress

We analyzed markers of gliosis and endoplasmic reticulum (ER) stress in the hypothalamus (Table 3). Gliosis (GFAP and Iba1) and ER stress markers (pJNK) were not altered in response to dietary intake (Table 3).

## 3. Discussion

As previously reported, long-term HFD consumption increased body weight, fat mass and circulating leptin levels in both sexes [42,43,44,45]. However, female mice needed more time before a significant weight gain was observed which is in concordance with previous observations in rodents [44,46], at least in young animals. Energy intake was also increased, particularly in females, with females on the HFD consuming more energy than males. Although both sexes gained significant weight, glucose tolerance was only affected in males, as previously shown after 16 weeks of HFD intake in C57/BL mice [45]. However, basal insulin levels and the HOMA index were increased in both sexes after HFD in agreement with previous studies [43].

Sex differences in the metabolic response to long-term dietary challenges have been previously reported [46,47,48]. Likewise, we and others have shown that the circulating IGF system also differs between male and female rodents, both at baseline [49,50] and in response to a short-term dietary change [39]. Male mice were found to have higher circulating levels of total IGF1, IGFBP2 and IGFBP3, whereas females have higher IGF2 levels, with no sex differences in free IGF1 levels, as previously reported in rats [50]. We previously reported higher levels of free and total IGF1, IGF2 and IGFBP3, but reduced IGFBP2 levels at baseline, in male compared to female rats [39]. The circulating IGF system has also been shown to differ between the sexes in humans [51,52]. Together, the sex differences in the growth hormone (GH)/IGF system in conjunction with sex steroids [53] underlie the differences in longitudinal growth between males and females.

In both male and female mice, the circulating IGF system was modified by HFD-induced obesity, similar to that observed in patients with obesity. In both sexes, circulating levels of free IGF1 [54], total IGF1, IGF2 [55,56,57] and IGFBP3 [58] are reported to be increased in humans and rodents with obesity, although some studies found no changes in total IGF1 in patients with obesity [54]. This general increase in the IGF system that is associated with obesity occurs despite GH hyposecretion, which contributes to adiposity [59,60]. Increased hepatic GH sensitivity most likely contributes to the maintenance of circulating IGF1 levels despite low GH secretion [61].

Rapid changes can be seen in the IGF system in response to a HFD even before weight gain is observed [39], suggesting that they may be caused by the diet per se. In contrast, the changes found here after long-term HFD consumption could be due to the modifications in overall nutritional status in addition to possible direct effects of the diet, whereas, in response to a short-term dietary change, the modifications observed in the IGF system were more abundant in response to CCD [39]—here, long-term effects were associated with HFD intake. This supports the hypothesis that changes associated with HFD intake are largely due to body weight gain.

Circulating IGFBP2, one of the most abundant IGFBPs in serum [62], is suggested to play a role in protection against HFD-induced obesity and diabetes onset [40,41]. A negative correlation between circulating IGFBP2 and body mass index (BMI) has been reported in obese patients [63] and, in children with obesity, IGFBP2 levels are reduced, whereas in anorexic adolescents they are increased [64]. We observed a negative correlation between IGFBP2 and body weight in male mice independently of the diet consumed, although the increase in IGFBP2 in male mice on a CCD was not associated with weight change. It is possible that changes in this binding protein are time-dependent and occur differently between males and females. Indeed, after one week of CCD IGFBP2 was decreased in female rats [39], while after a more long-term dietary change, this binding protein was not different from those on a chow diet. The augmented IGFBP2 in males after more long-term CCD could be an attempt to protect against the higher sucrose intake. The susceptibility to diet-induced obesity and its metabolic complications differs between the sexes [46,65] and this could be related to the differential changes in IGFBP2. However, more studies are clearly needed for a better comprehension of the role of IGFBP2 in metabolism.

IGF1 is important for adipocyte metabolism, maturation and differentiation [19,66,67]. In male C57 mice, IGF1 mRNA levels were reported to be decreased in adipocytes purified from perigonadal adipose tissue of obese compared to lean mice, despite the lack of change in IGF1 expression in lysates of this same tissue [68]. This could suggest that although IGF1 mRNA levels may be reduced in adipocytes, other cell types in adipose tissue, such as macrophages, could increase their expression of *Igf1* such that global IGF1 mRNA levels in VAT do not change. Here, IGF1 mRNA levels were reduced in VAT after HFD in males, with the possibility that adipocytes in VAT decrease their IGF1 mRNA levels after HFD intake with the VAT macrophages or circulating IGF1 providing the source of IGF1 required to maintain adipocyte hypertrophy and hyperplasia [68]. This system is not altered in females, which are metabolically less affected by HFD consumption despite the increase in the VAT percentage observed in response to a HFD intake. If a longer exposition to this diet is required to alter it in females, or if this system is sex-dependent, deserves further study. As seen here, IGF2 mRNA levels in VAT were previously reported to be reduced in C57 mice in response to HFD-induced obesity; however in PWK mice, which are resistant to HFD-induced obesity, they are not [69]. These authors suggest that IGF2 has an anti-inflammatory effect on TNF-α-induced MCP-1 expression in adipocytes [69]. They also reported increased expression of IGF2R in VAT in HFD-exposed C57 mice [69], which was not found here possibly due to the shorter exposure to the HFD in our study.

During adipogenesis, IGFBP2 is the principal IGFBP produced by visceral white adipocytes, inhibiting both adipogenesis and lipogenesis [70], with levels reported to be reduced in obese *ob/ob*, *db/db* and high-fat-fed mice [71]. This reduction was accompanied by decreased IGFBP2 in circulation, suggesting that the decreased levels in VAT after HFD intake may lead to a decrease in circulating IGFBP2 levels [71]. Here we found no effect of HFD on IGFBP2 mRNA levels in VAT or circulating IGFBP2; again, it is possible that a longer exposition to a HFD may be needed.

In PAPP-A knockout mice, adipocyte size and lipid uptake are reduced in mesenteric fat, and visceral fat did not expand in PAPP-A KO mice exposed to a HFD, suggesting that the local cleavage of IGFBP4 is mandatory for adipocyte expansion [72]. Local free IGF1 levels are suggested to increase in adipose tissue in response to PAPP-A secretion in pregnant women [73], supporting the idea that IGF1 may contribute to the adipocyte hypertrophy associated with HFD intake through PAPP-A activity, and the reduction in these factors could be a negative feedback effect in attempt to block excess expansion. However, we found no effect of diet on PAPP-A2 expression, although males had significantly higher levels than females. Whether this protease is involved in sex differences in VAT function remains to be determined.

Both the CCD and HFD reduced STC-1 mRNA levels in male mice. In cultured adipocytes from male rats, STC-1 was shown to increase glucose uptake and the storage of triacylglycerol during the postprandial period [74]. Thus, this reduction in STC-1 mRNA levels may be a response conducted to control the lipid accumulation by VAT and this could possibly be induced by the higher amount of fat content in both the CCD and HFD compared to the chow diet. STC-2 mRNA levels were not altered in VAT, but the possible role of STCs regulating the IGFs availability in VAT, and potentially modifying the adipocyte size, deserves further research.

Hypothalamic IGF1 mRNA levels were increased after HFD consumption in both sexes, in agreement with our previous results in male mice after 7 weeks of HFD [75]. This increase could be a homeostatic mechanism to counteract the central effects of a HFD as increased central expression of IGF1 has been shown to improve glucose tolerance and insulin sensitivity [76]. Hypothalamic IGF2 mRNA levels were also increased after HFD, especially in females. Maternal HFD was shown to increase hypothalamic IGF2 mRNA levels in female Sprague Dawley rat offspring at postnatal day 10, with no changes in males [77], but little is known regarding central IGF2 in metabolism. In the adult brain, IGF2 promotes memory consolidation, neurogenesis and cognitive function [78] and it could be involved in structural changes induced by HFD and/or obesity, although this remains to be determined.

Hypothalamic levels of IGF2 and IGFBP2 were positively correlated, as previously reported in rats [39]. In females, both IGF2 and IGFBP2 were positively correlated with glycemia. A role of both IGFBP2 and IGF2 in glucose metabolism has been reported [41,79], but whether these factors participate in central glucose regulation remains unknown. The fact that females were metabolically less affected by long-term HFD consumption compared to males could suggest a possible metabolic effect of these factors at the level of the hypothalamus.

The role of IGFBP5 in metabolism has been poorly studied, particularly at the central level. Male IGFBP5-deficient mice are reported to have a greater body weight, impaired glucose tolerance at baseline and a larger increase in adipose tissue on a HFD compared to wild type [80]. This suggests that IGFBP5 is protective against obesity and glucose metabolism impairment, but the specific role of hypothalamic IGFPB5 in metabolic control is unknown. As this binding protein is increased after both HFD and CCD consumption in males one might speculate that it could be a protective response against these diets at the central level, and that this could be induced by the higher fat content in both of these diets compared to chow. No other members of the IGF system analyzed in the hypothalamus were affected by either diet or sex.

No evidence of gliosis or ER stress was found in the hypothalamus. Although various authors have reported both phenomena as well as central inflammation after 8 weeks of HFD consumption [81,82,83] or even earlier [81], other authors report no alterations in hypothalamic inflammation in male mice [84]. Thus, the causal relationship between hypothalamic inflammation and gliosis in weight gain is unclear [85]. In addition, the absence of gliosis observed in our study in response to HFD is accompanied by an increase in the gene expression of IGF1 and 2, which may play a neuroprotective role by reducing cell stress [27].

Diet composition is reported to be more important than caloric intake per se in determining NPY/AgRP neuron activity [86] and NPY and AgRP were reduced in the hypothalamus after HFD consumption in both sexes, as previously reported in male rats [75,87,88]. This is most likely related to the satiating properties of the HFD inducing a response that attempts to reduce the amount of energy consumed. However, in females this response is attenuated, at least regarding NPY, and this could underlie their higher energy intake compared to both HFD males and control females. Insulin decreases both NPY and AgRP expression in the hypothalamic neuronal line mHypoE-46 [89]. Considering the insulin-like actions of IGF1 and 2, it is possible that the rise in these factors with HFD intake contributes to the inhibition of orexigeneic neuropeptide expression. Administration of IGF icv has been shown to increase POMC mRNA levels in chicks [90], which was not observed here. This difference could be due to the difference in species. An overall effect of HFD to increase the anorexic neuropeptide CART was found, but no changes were observed in POMC expression. On the contrary, we previously found POMC mRNA levels to increase after HFD in male mice with no effect on CART relative expression [75], whereas other authors reported no significant changes in the hypothalamic POMC and CART mRNA expression in female mice on a long-term HFD [91], employing a different source of HFD (45% kcal from fat, 4.73 kcal/g). In our previous study [75], the exposition to HFD was for only 50 days instead of the 8 weeks here, which may explain some of these differences.

The CCD used here is what has been traditionally referred to as a control low-fat diet (LFD) as it was developed to be used as the control to the commercial HFD. However, it is now clear that these commercially developed control diets can also have metabolic effects as their nutrient composition differs from a normal chow diet. Here, the CCD did not modify body weight, but basal insulin levels and HOMA index were increased in both sexes. Although the caloric content is similar between the rodent chow and CCD, the amount of sucrose, 33% in CCD and 0.9% in chow, differs considerably. Dry sucrose or polycose consumption does not affect final body weight in rats [92,93], nor does consumption of a 33% sucrose solution, despite the metabolic alterations which are found [94,95]. However, these studies found fat mass and specific metabolic parameters to be modified. Thus, it is possible that the high sucrose composition in the CCD induces these observed metabolic alterations, again indicating that not only caloric intake, but dietary composition, is important.

The response of the IGF system to CCD intake is time- and sex-dependent, whereas, in female rats exposed to 1 week of CCD, IGFBP2 levels were reduced [39] and no changes were found in this sex after 8 weeks. In contrast, males showed no differences at one week of CCD intake [39] but had increased serum IGFBP2 levels after 8 weeks. This sex-specific response may be related to the protective role of IGFBP2 in glucose metabolism [40,41] as glucose metabolism is differently affected in males and females. Short- and a long-term HFD intake also differently affected IGF2 levels in males. Some authors suggest that an initial reduction in serum IGF2 levels may indicate a bad prognosis for weight gain [96], whereas longer HFD exposition leads to an increase in the circulating IGF2 as in obesity [56]. What is clear is that each diet has differential metabolic implications that are time- and sex-dependent.

In conclusion, long-term HFD intake modulates the IGF system in a tissue-dependent manner, which may respond to different tissue requirements, and suggests an active role of this system in the metabolic response to this dietary challenge. Moreover, most of the observed changes are sex-specific and this could be involved in the differential metabolic responses between males and females.

## 4. Materials and Methods

### 4.1. Ethical Statement

This study was designed following the European Communities Council Directive (2010/63/UE) and the Spanish Royal Decree 53/2013 concerning the protection of experimental animals. In addition, the study was approved by the Ethical Committee of Animal Experimentation of the Hospital Puerta de Hierro de Madrid and the Animal Welfare Organ of the Comunidad Autónoma de Madrid (07/346225.9/15, 9 April 2015). All care was taken to use the minimum number of animals.

### 4.2. Animals and Diets

In this study, 54 seven-week-old C57BL/6J mice (27 males and 27 females) were purchased from Charles River Laboratories. Upon arrival, animals were weighed and randomly distributed into cages according to sex (3 mice per cage) and allowed to acclimate for a week with free access to a normal chow diet and tap water. The mice were weighed again and randomly distributed into the different experimental groups. They were fed ad libitum with a high-fat diet (HFD; 62% kcal from fat, 18% kcal from proteins, 20% kcal from carbohydrates, 5.1 kcal/g, LabDiet), a commercial control diet (CCD; 10% kcal from fat, 18% kcal from proteins, 72% kcal from carbohydrates, 3.76 kcal/g, LabDiet) or standard rodent chow (6% kcal from fat, 17% kcal from proteins, 77% from carbohydrates, 3.41 kcal/g, Panlab) for 8 weeks, with free access to tap water throughout the study. This resulted in a total of 6 groups with nine animals per diet and sex (*n* = 9). Body weight and food intake were monitored each week. Animals were maintained at 22 ± 2 °C throughout the study.

### 4.3. Glucose Tolerance Test (GTT)

A week before sacrifice, six mice per group (*n* = 6) were fasted for 6 h prior to testing. They were weighed and then intraperitoneally injected with 2 mg of D-glucose per gram of body weight. Glycemia was determined in a drop of blood from the tail by using a Freestyle Optimum Neo glucometer (Abbott, Witney, UK). Glycemia was measured just prior to the injection (basal, 0 min) and at 30, 60, 90 and 120 min after the D-glucose injection.

### 4.4. Tissue Collection

Mice were weighed and fasted 12 h before sacrifice by decapitation, which took place between 9:00 and 11:00 am. A few days before sacrifice, sawdust from male cages was mixed in the females’ cage to equalize estrous cycle in females, which was determined by vaginal cytology at sacrifices. In total, 18.5% of the females were on proestrus phase, 77.8% were in estrus, 0% in metaestrus and 3.7% in diestrus. Trunk blood was collected in tubes containing a 0.5 M ethylenediaminetetraacetic acid (EDTA) solution. Samples were then centrifuged at 3000 rpm for 15 min at 4 °C and plasma was collected and aliquoted (to avoid freeze–thaw cycles) and stored at −80 °C until used. Perigonadal visceral adipose tissue (VAT) was dissected, weighted and frozen at −80 °C. The hypothalamus (rostrally limited by the optic chiasm and caudally by the mammillary bodies) was also dissected and kept at −80 °C until processed.

### 4.5. ELISA Assays

Plasma levels of free IGF1 (AnshLabs, Webster, TX, USA), total IGF1 (Mediagnost, Reutlingen, Germany), IGF2 (R&D Systems, Minneapolis, MN, USA), IGFBP2 (Millipore, Burlington, MA, USA), IGFBP3 (Mediagnost), insulin (Millipore) and leptin (Millipore) were measured following the manufacturers’ instructions. Absorbance was read by spectrophotometry (Tecan Infinite M200, Grödig, Austria).

Homeostatic Model Assessment for Insulin Resistance (HOMA-IR) was calculated by using the following equation: HOMA-IR = glycemia (mmol/L) × insulin (mU/L)/22.5

### 4.6. RNA and Protein Extraction

An RNeasy Lipid Tissue Mini Kit (Qiagen, Hilden, Germany) was used for visceral adipose tissue RNA extraction according to manufacturer’s instructions, whereas an RNeasy Plus Mini Kit (Qiagen) was used following the manufacturer’s instructions for hypothalamic RNA isolation. Protein was extracted from the eluate after tissue lysis. The eluate was mixed with 4 volumes of cold acetone and stored overnight at −20 °C. The samples were centrifuged 10 min at 3000 rpm and the pellets containing the protein were resuspended in a CHAPS hydrate (Sigma-Aldrich, Saint Louis, MO, USA) solution, containing 7 M urea, 2 M thiourea, 4% CHAPS, 0.5% 1M Tris pH 8.8 in distilled water and stored at −80°C. For protein quantification, Protein Assay Dye Reagent Concentrate (Bio-Rad Laboratories, Hercules, CA, USA) was used to perform the Bradford assay.

### 4.7. Western Blotting

Depending on the expected signal and molecular weight of each target, 10 to 40 µg of protein were resolved on 8, 10 or 12% sodium dodecyl sulphate-denaturing polyacrylamide gels. After electrophoresis, proteins were transferred to a previously activated polyvinylidine difluoride (PVDF) membrane and then blocked with a Tris-buffered saline buffer containing 0.1% Tween 20 and 5% non-fat dried milk or bovine serum albumin (BSA) when phosphorylated proteins were assayed. Primary antibodies (Table 4) were diluted in the same buffer and incubated O/N with agitation at 4 °C. The next day, the corresponding horseradish peroxidase-conjugated secondary antibody was diluted in the same buffer and the membranes incubated for 1.5 h. Clarity Western ECL Substrate (Bio-Rad Laboratories, Hercules, CA, USA) was employed to visualize the peroxidase activity and the chemiluminescent signal was detected by using ImageQuant Las 4000 Software (GE Healthcare Life Sciences, Barcelona, Spain). To normalize for protein loading, GAPDH was used, as indicated.

### 4.8. Real-Time qPCR

For RT-qPCR, RNA (0.5–1 µg) was retro-transcribed to copy DNA (cDNA) by using an NZY First-Strand cDNA Synthesis Kit (NZYTech, Lisbon, Portugal). TaqMan probes of target genes (Table 5) were used for qPCR in a QuantStudio 3 Real-Time PCR System (Applied Biosystems, Carlsbad, CA, USA). Mouse glyceraldehyde 3-phosphate dehydrogenase (GAPDH) endogenous control (Applied Biosystems) was employed as the housekeeping gene, except on visceral adipose tissue, for which *Ppia* was chosen (Table 5). For the mathematical analysis, the ΔΔCT method was performed. Relative levels of expression are expressed as percentage of the chow male group.

### 4.9. Statistical Analysis

Statistics analyses were performed using SPSS 15.0 (SPSS Inc., Chicago, IL, USA) software. A two-way ANOVA was employed using the diet and sex factors. Weight gain and food intake over time were calculated by a repeated measures ANOVA, as well as the weekly data for these variables. For the glucose tolerance test, the area under the curve (AUC) was calculated by using GraphPad Prism 5 software (San Diego, CA, USA). Data are presented as the mean ± standard error of the mean (SEM). Graphs were made by GraphPad Prism 5 software. For the linear correlation between variables, a Pearson correlation coefficient was calculated. *p <* 0.05 was considered significant.

## Figures and Tables

**Table 1:** Body weight, fat mass, glycemia, energy intake and circulating leptin levels in male and female mice exposed for 8 weeks to a high-fat diet (HFD), commercial control diet (CCD) or a chow diet. a: effect of sex, b: effect of diet, c: interaction between sex and diet, #: different from chow on the same sex, @: differences between sexes on the same diet, *n* = 6, except energy intake was *n* = 3 (number of cages/group).

	Chow Males	CCD Males	HFD Males	Chow Females	CCD Females	HFD Females	Significance
Final body weight (g)	24.6 ± 0.5	26.4 ± 0.3	35.2 ± 1.0 ^#^	19.6 ± 0.3 ^@^	20.3 ± 0.3 ^@^	24.2 ± 1.5 ^#,@^	a, *p <* 0.001 b, *p <* 0.001 c, *p <* 0.01
Visceral adipose tissue (%)	1.16 ± 0.07	1.22 ± 0.14	4.31 ± 0.28 ^#^	0.62 ± 0.07 ^@^	0.99 ± 0.12	2.19 ± 0.46 ^#,@^	a, *p <* 0.001 b, *p <* 0.001 c, *p <* 0.001
Subcutaneous adipose tissue (%)	0.52 ± 0.04	0.57 ± 0.05	1.86 ± 0.16 ^#^	0.67 ± 0.04 ^@^	0.96 ± 0.09 ^@^	1.52 ± 0.21 ^#,@^	b, *p <* 0.001 c, *p <* 0.001
Glycemia (mg/dl)	67.8 ± 2.0	71.8 ± 3.7	80.6 ± 3.2	63.6 ± 3.9	68.7 ± 4.3	79.6 ± 4.9	b, *p <* 0.01
Kcal/mouse/ day	11.7 ± 0.1	10.9 ± 0.8	13.8 ± 0.5	10.0 ± 0.2 ^@^	9.9 ± 0.2	24.9 ± 0.4 ^#,@^	a, *p <* 0.001 b, *p <* 0.001 c, *p <* 0.001
Kcal/mouse/ day/100 g	46.2 ± 0.7	42.3 ± 2.0	45.8 ± 0.7	48.7 ± 1.2	47.6 ± 0.9	114.5 ± 3.3 ^#,@^	a, *p <* 0.001 b, *p <* 0.001 c, *p <* 0.001
Energy efficiency (%)	0.68 ± 0.02	0.96 ± 0.11	1.84 ± 0.06 ^#^	0.56 ± 0.02 ^@^	0.50 ± 0.05 ^@^	0.45 ± 0.07 ^@^	a, *p <* 0.001 b, *p <* 0.001 c, *p <* 0.001
Leptin (ng/mL)	0.77 ± 0.28	0.65 ± 0.21	10.05 ± 2.10 ^#^	1.06 ± 0.34	1.22 ± 0.49	4.52 ± 1.43 ^#^	b, *p <* 0.01 c, *p <* 0.05

**Table 2:** Linear correlation between circulating levels of IGF2 and IGFBP2, relative mRNA levels of IGF2 and IGFBP2 in visceral adipose tissue (VAT) and in the hypothalamus with body weight and glycemia in both sexes. * *p <* 0.05, ** *p <* 0.01, *n* = 6.

	Plasma IGF2	Plasma IGFBP2	VAT IGF2 mRNA	VAT IGFBP2 mRNA	HPT IGF2 mRNA	HPT IGFBP2 mRNA
Body weight (males)	0.661 **	−0.470 *	−0.523 *	−0.306	0.088	0.090
Body weight (females)	0.286	−0.225	−0.107	−0.132	0.579 **	0.598 **
Glycemia (males)	0.159	−0.102	−0.218	0.075	−0.092	0.138
Glycemia (females)	0.268	−0.016	−0.157	−0.293	0.570 **	0.623 **

**Table 3:** Effects of 8 weeks on a high-fat diet (HFD), commercial control diet (CCD) or chow diet on glial structural protein (GFAPs: Glial fibrillary acidic protein, Iba1: Ionized calcium binding adaptor molecule 1) and endoplasmic reticulum (ER)-stress markers (pJNK: phosphorylated c-Jun N-terminal kinases). ns = non-significant, *n* = 6.

	Chow Males	CCD Males	HFD Males	Chow Females	CCD Females	HFD Females	Significance
**GFAP**	100.0 ± 2.1	115.6 ± 6.8	107.4 ± 9.5	103.9 ± 7.6	97.9 ± 3.0	103.3 ± 4.5	**ns**
**Iba1**	100.0 ± 3.2	117.1 ± 3.6	110.4 ± 12.5	114.8 ± 7.4	101.5 ± 4.5	97.7 ± 6.9	**ns**
**pJNK**	100.0 ± 10.8	84.0 ± 11.5	71.6 ± 5.2	107.5 ± 18.3	86.7 ± 13.9	95.3 ± 14.1	**ns**

**Table 4:** Antibodies used for Western blotting.

Antibody	Class	Dilution	Host	Commercial Source	Reference
pJNK	Polyclonal	1:3000	Rabbit	Promega	V7932
GAPDH	Polyclonal	1:10,000	Rabbit	Sigma-Aldrich	G9545
GFAP	Polyclonal	1:5000	Guinea pig	Synaptic Systems	173004
Iba1	Polyclonal	1:1000	Rabbit	Synaptic Systems	234003
α-guinea pig HRP-conjugated	Polyclonal	1:2000	Goat	AbD Serotec	AHP861P
α-rabbit HRP-conjugated	Polyclonal	1:20,000	Goat	Invitrogen	31460

**Table 5:** List of TaqMan probes used for qPCR.

Name	Gene	Reference	Commercial Source
Agouti-related protein	*Agrp*	Mm00475829_g1	Applied Biosystems
Cyclophilin A (Peptidylprolyl isomerase A)	*Ppia*	Mm02342430_g1	Applied Biosystems
Cocaine and amphetamine regulated transcript prepropeptide	*Cartpt*	Mm04210469_m1	Applied Biosystems
Insulin-like growth factor 1	*Igf1*	Mm00439560_m1	Applied Biosystems
Insulin-like growth factor 1 receptor	*Igf1r*	Mm00802831_m1	Applied Biosystems
Insulin-like growth factor 2	*Igf2*	Mm00439564_m1	Applied Biosystems
Insulin-like growth factor 2 receptor	*Igf2r*	Mm00439576_m1	Applied Biosystems
Insulin-like growth factor-binding protein 2	*Igfbp2*	Mm00492632_m1	Applied Biosystems
Insulin-like growth factor-binding protein 3	*Igfbp3*	Mm01187817_m1	Applied Biosystems
Insulin-like growth factor-binding protein 4	*Igfbp4*	Mm00494922_m1	Applied Biosystems
Insulin-like growth factor-binding protein 5	*Igfbp5*	Mm00516037_m1	Applied Biosystems
Neuropeptide Y	*Npy*	Mm03048253_m1	Applied Biosystems
Pregnancy-associated plasma protein A	*Pappa*	Mm01259244_m1	Applied Biosystems
Pregnancy-associated plasma protein A-2	*Pappa2*	Mm01284029_m1	Applied Biosystems
Pro-opiomelanocortin	*Pomc*	Mm00435874_m1	Applied Biosystems
Stanniocalcin-1	*Stc1*	Mm01322191_m1	Applied Biosystems
Stanniocalcin-2	*Stc2*	Mm00441560_m1	Applied Biosystems
